# Kansas City Dental College

**Published:** 1893-07

**Authors:** 


### EDITORIAL.

### Kansas City Dental College.

The Kansas City Dental College has recently purchased the ground and buildings at the corner of Tenth Street and Troost Avenue, Kansas City, Mo., which will at once be remodeled to suit every requirement of a first-class Dental college, and will be ready for occupation at the opening of the next session.

We congratulate the members of the Association, the officers and faculty, that they are now in possession of a building of their own, in which there will be ample and suitable accommodation for the various departments connected with the College, and wish them success in their new quarters.

The fourth edition of "Letters from a Mother to a Mother, by Mrs. M. W. J., has just been issued by the Wilmington Dental Manufacturing Co., of Philadelphia. Of it we will make a fuller mention in next issue.

The History of the National Association of Dental Faculties is just from the press. This is a very important document, and especially for those interested in Dental College work.

It can be obtained by addressing Dr. J. E. Cravens, Indianapolis, Ind.

